# ASSOCIATION BETWEEN LONG-TERM CARE FACILITY STAFF TRUST IN LEADERSHIP AND RECEIVING THE COVID-19 VACCINE

**DOI:** 10.1093/geroni/igad104.3191

**Published:** 2023-12-21

**Authors:** Xinjun Li, David Gifford, Valerie Williams

**Affiliations:** AHCA/NCAL, Washington, District of Columbia, United States; AHCA/NCAL, Washington, District of Columbia, United States; AHCA/NCAL, Washington, District of Columbia, United States

## Abstract

Trust in the sources recommending COVID-19 vaccine is thought to be associated with COVID-19 vaccine uptake. We compared long-term care (LTC) staff’s trust with leadership and public health organizations and their vaccination status. We administered a questionnaire to all staff in 48 long term facilities (39 Skilled Nursing Facilities (SNFs) and 9 Assisted Living (AL) facilities) to assess their overall trust level on a 5-point Likert scale with leadership and public health agencies along with their reported used COVID-19 vaccination status. We received 759 responses, with 34 were excluded due to incomplete data. Overall, 12% were not vaccinated, 25.2% completed initial series, 25.0% received one booster, and 37.8% received bivalent booster. Average trust was lowest with CDC (3.62) & CMS (3.60) and highest with leadership (Supervisor (4.07), Administrator (3.98) and DON (3.89)), which were higher than trust with the LTC organization (3.74). Trust levels increased as the staff’s vaccination status increased (p< 0.01) for each category in ANOVA tests. Building overall trust may help increase staff compliance with vaccination recommendations.

